# Transformational Leadership and Organizational Citizenship Behavior: A Meta-Analytic Test of Underlying Mechanisms

**DOI:** 10.3389/fpsyg.2017.01364

**Published:** 2017-08-10

**Authors:** Christoph Nohe, Guido Hertel

**Affiliations:** Department of Psychology, Organisational and Business Psychology, University of Münster Münster, Germany

**Keywords:** transformational leadership, organizational citizenship behavior, affective organizational commitment, job satisfaction, trust in the leader, leader-member exchange

## Abstract

Based on social exchange theory, we examined and contrasted attitudinal mediators (affective organizational commitment, job satisfaction) and relational mediators (trust in leader, leader-member exchange; LMX) of the positive relationship between transformational leadership and organizational citizenship behavior (OCB). Hypotheses were tested using meta-analytic path models with correlations from published meta-analyses (761 samples with 227,419 individuals overall). When testing single-mediator models, results supported our expectations that each of the mediators explained the relationship between transformational leadership and OCB. When testing a multi-mediator model, LMX was the strongest mediator. When testing a model with a latent attitudinal mechanism and a latent relational mechanism, the relational mechanism was the stronger mediator of the relationship between transformational leadership and OCB. Our findings help to better understand the underlying mechanisms of the relationship between transformational leadership and OCB.

## Introduction

Over the past three decades, transformational leadership has emerged as one of the predominant paradigms to understand leadership effectiveness (Avolio et al., 2009). Transformational leadership theory is based on the notion that certain leader behaviors transform followers' values, needs, preferences, and aspirations, and motivate them, “to perform above and beyond the call of duty” (House et al., 1991, p. 364). One important construct that captures follower performance beyond the call of duty is organizational citizenship behavior (OCB), referring to discretionary extra-role behavior enhancing the organizational environment that supports task performance (Organ, 1997). In today's complex and fast-paced organizations, employee extra-role behavior that goes beyond limited lists of job duties have become crucial for organizational success (Organ et al., 2006). Indeed, high OCB is associated with high customer satisfaction, low turnover, and even with high in-role performance (Podsakoff et al., 2009). In the leader-follower relationship, OCB is perhaps best suited to reflect follower performance consequences because engaging in or withholding voluntary extra-role behaviors is a more flexible and saver means to repay leader treatment compared to task performance (Organ, 1988). Although, empirical evidence consistently supports positive correlations between transformational leadership behaviors and OCB (Wang et al., 2011; Carter et al., 2014), the psychological mechanisms underlying this relationship are less clear.

Social exchange theory (Blau, 1964) is probably the most influential framework for explaining the general dynamics from which OCB emerge. Drawing on this framework, two specific psychological mechanisms through which transformational leadership behaviors elicit employee OCB can be distinguished. The first mechanism is based on the assumption that transformational leadership positively affects employees' general job attitudes, such as organizational commitment or overall job satisfaction (e.g., Judge and Piccolo, 2004), which in turn contribute to more OCB (e.g., Ilies et al., 2009). This explanation considers transformational leadership effects on OCB as mere *reactions* of followers to positive leadership behaviors. However, transformational leadership effects might also include more complex (bi-directional) relational exchange processes between leaders and followers that evolve over time. A second explanation of the relationship between transformational leadership and OCB stresses such relational processes between leaders and followers. According to this perspective, transformational leaders and followers engage in a high-quality relationship characterized by, for example, trust and leader-member exchange (LMX; Dirks and Ferrin, 2002; Dulebohn et al., 2012). Consequently, followers of transformational leaders engage in more OCB to reciprocate good leader treatment (Wang et al., 2005).

Although considerable research addressed the relational mechanism (e.g., Wang et al., 2005; Burke et al., 2007; Zhu et al., 2013), we are not aware of studies examining the attitudinal mechanism or the relational and attitudinal mechanisms in concert (for an exception see Podsakoff et al., 1990). Thus, little is known about the theoretically relevant but largely neglected attitudinal process and about the relative strength of relational vs. attitudinal mechanisms. Based on social exchange theory (Blau, 1964), the present study addresses this issue and examines two mechanisms of the transformational leadership—OCB relationship: mere attitudinal reactions of employees to leader behaviors (i.e., job satisfaction and affective organizational commitment) and more complex bi-directional relationships between leader and followers characterized by interactivity and reciprocity (i.e., trust in the leader and leader-member exchange; LMX). Additionally, we test whether the attitudinal and the relational mechanisms are equally strong in mediating the transformational leadership—OCB relationship or whether they differ in magnitude. To test these assumptions, we used meta-analytic path models with correlations from published meta-analyses. Thus, this study used a broad data base to examine and contrast two theoretically derived mechanisms of the relationship between transformational leadership behaviors and OCB.

The present study contributes to the leadership literature in several important ways. First, by simultaneously considering job satisfaction, affective organizational commitment, trust in the leader, and LMX as potential mediators of the relationship between transformational leadership and OCB, this study can test the relative strength of those four mediators and can provide insights about whether attitudinal or relational mechanisms dominate the transformational leadership—OCB relationship. Relatedly, by simultaneously including those four potential mediators, the current study provides a more complete picture of mechanisms underlying the relationship between transformational leadership and OCB compared to prior studies that tested single mediators only, such as trust (Zhu et al., 2013) or LMX (Wang et al., 2005).

Second, by using correlations from published meta-analyses, our results are based on a broader databases compared to previous studies that tested potential mediators with smaller sample sizes (e.g., Podsakoff et al., 1990). Thus, the concern of a low generalizability applies to a much lesser extent to our results compared to prior studies using single samples. Notably, two recent meta-analyses addressed mechanisms of the relationship between transformational leadership and performance-related outcomes (Gottfredson and Aguinis, 2017; Ng, 2017). However, those two meta-analyses failed to address trust in the leader as mediator of the relationship between transformational leadership and OCB. This omission seems unfortunate, given that trust in the leader is an important aspect of models of transformational leadership research (Braun et al., 2013). We contribute to the literature by examining trust in the leader as mediator of the relationship between transformational leadership and OCB, and by contrasting trust in the leader with attitudinal mediators. Additionally, our study goes above and beyond Ng (2017) and Gottfredson and Aguinis (2017) by testing the attitudinal and the relational mechanisms on a higher-order level with latent mediators. Thereby, our study illuminates those two mechanisms focusing on a more theoretical level instead of single constructs.

Finally, research on transformational leadership behaviors and potential outcomes has been criticized for studying a diverse set of mediators without a clear theory that guides the examination of mediation (Van Knippenberg and Sitkin, 2013). Drawing on social exchange theory as a theoretical framework, the current study responds to this critique by considering a theoretically coherent set of mediating variables for the relationship between transformational leadership and OCB. Thereby, this study also works toward integrating the previously segmented study of mediators of the relationship between transformational leadership behaviors and OCB.

### Transformational leadership, social exchange theory, and OCB

In the original conceptualization (Burns, 1978; Bass, 1985), transformational leadership includes four dimensions of leader behavior. *Idealized influence* refers to the degree to which leaders show admirable behaviors which cause followers to identify with them. *Inspirational motivation* refers to the degree to which leaders articulate visions that are appealing and inspiring to followers. *Intellectual stimulation* refers to the degree to which leaders take risks, challenge assumption, and solicit followers' ideas. *Individual consideration* refers to the degree to which leaders listen to followers' concerns, attend to their needs, and act as mentors or coaches. Although, researchers consider transformational leadership behaviors important for achieving high employee in-role performance, they propose that such leadership behaviors are even more important for achieving high extra-role performance such as OCB (Podsakoff et al., 1996). Indeed, OCB might be particularly sensitive for leadership behavior transmitting more general goals or visions that include unplanned voluntary activities of followers, whereas in-role performance might be better suited to reflect specific goal setting or transactional leadership.

Social exchange theory is probably the most influential theory for explaining the general dynamics from which OCB emerge and provides a framework to understand why transformational leadership behaviors are associated with more follower OCB (Wang et al., 2005). In his seminal work, Blau (1964) distinguished between economic and social exchange relationships. Economic exchange is contractual in that it involves the exchange of specified terms and tangible resources. Social exchange, however, relates to vaguely specified obligations and involves the exchange of less tangible resources. Social exchange relationships are characterized by loyalty and trust, and evolve over time when parties follow the norm of reciprocity (Cropanzano and Mitchell, 2005). The distinction between economic and social exchange is similar to the distinction between transactional and relational contracts made by Rousseau and Parks (1993). They describe contracts as agreements that create an obligation to do or not do something. On one end of the continuum are transactional contracts, which are “short-term monetizable agreements with limited involvement of each party in the lives and activities of the other” (Rousseau and Parks, 1993, p. 10). On the other end of the continuum are relational contracts, which are typically open-ended, long-term contracts that comprise the exchange of socio-emotional elements. Whereas, transactional contracts parallel the notion of economic exchange, relational contracts parallel the notion of social exchange (Konovsky and Pugh, 1994).

In contrast to transactional leaders who offer their followers a proper exchange of tangible resources and are likely to engage in economic exchange relationships, transformational leaders offer a purpose that focuses on higher order intrinsic needs and transcends short-term interests (Judge and Piccolo, 2004). According to this perspective, employees can engage in a social exchange relationship with their transformational leader and reciprocate his/her behaviors by engaging in OCB. Based on the general principles of social exchange theory, scholars used several different measures to capture the existence of social exchange processes. In their review on social exchange theory, Cropanzano and Mitchell (2005) advocated LMX, affective commitment, trust, and perceived support as measures of social exchange mechanisms. Additionally, Ilies et al. (2009) used social exchange theory to argue that increased job satisfaction influences employees to engage in more OCB. Building on those prior studies, we used job satisfaction and affective organizational commitment as a bundle of attitudinal indicators and LMX and trust as a bundle of relational indicators. Although the mechanisms selected for the present study are unified by social exchange theory, comparisons among them are warranted because the mechanisms reflect conceptually distinct phenomena. Job satisfaction and affective organizational commitment reflect fundamental evaluations of employees' job experiences (Harrison et al., 2006), whereas trust in the leader and LMX reflect interpersonal, dyadic relationships in the context of organizations. We opted against perceived supervisor support, because it reflects employees' perceptions of leader behaviors leading to potential problems of multicollinearity with transformational leadership behaviors. Thus, building on social exchange theory, two specific psychological mechanisms through which transformational leadership behaviors elicit employee OCB can be distinguished: an attitudinal mechanism reflecting mere reactions of followers to leader behaviors and a relational mechanism reflecting more complex bi-directional relationships between followers and leaders.

### Transformational leadership behaviors and OCB: an attitudinal mechanism

Positive job attitudes (i.e., job satisfaction and affective organizational commitment) can at least in part explain the relationship between transformational leadership behaviors and OCB. Although prior research rarely tested this assumption (for an exception see Podsakoff et al., 1990), it follows from combining research on the antecedents of OCB with models of the effects of transformational leadership. In the following, we explain our reasoning in more detail. Job satisfaction can be defined as “the pleasurable emotional state resulting from the appraisal of one's job as achieving or facilitating the achievement of one's job values” (Locke, 1969, p. 316). Transformational leaders are likely to contribute to high levels of job satisfaction by giving followers personal attention on a one-on-one basis, learning each follower's needs, and attempting to address them (Walumbwa et al., 2005). Additionally, transformational leaders can influence how followers perceive their core job characteristics in terms of high significance, autonomy, variety, identity, and feedback (Piccolo and Colquitt, 2006). Consequently, followers will be more satisfied with their jobs (Loher et al., 1985).

Researchers have proposed a positive relationship between job satisfaction and OCB ever since the construct of OCB was introduced to the literature. An explanation of the positive relationship between job satisfaction and OCB argues that people are more likely to show OCB when they are in a positive mood. George and Brief (1992) suggested several arguments for this notion. For example, people in a positive mood help others because they want to maintain their good mood or because positive mood evokes mood congruent information making people feel more positively toward others. Given that job satisfaction at least partially captures positive moods in the workplace, it is more likely that employees show OCB when they experience high levels of job satisfaction (McNeely and Meglino, 1994). A more recent explanation of this relationship is based on social exchange theories. According to this perspective, employees view job satisfaction as a positive outcome of a social exchange relationship, and they reciprocate by engaging in OCB (Ilies et al., 2009). From the theory and research presented above follows that job satisfaction should at least partially mediate the positive relationship between transformational leadership and OCB. We propose:

*Hypothesis 1: Job satisfaction partially mediates the positive relationship between transformational leadership behaviors and OCB*.

In addition to job satisfaction, affective organizational commitment is a second attitudinal mechanism that can explain the transformational leadership—OCB relationship. Affective organizational commitment is defined as “an emotional attachment to, identification with, and involvement in the organization” (Meyer et al., 2002, p. 21). Shamir et al's (1993) self-concept based theory suggests that transformational leaders can facilitate followers' affective organizational commitment by engaging followers' self-concept in the interest of the leader's mission. Specifically, transformational leaders offer attractive and compelling goals for the organization's future and make the linkages between follower effort and organizational goal achievement more salient. As a result, followers should internalize organizational goals and act in the interest of goal accomplishment. In line with this reasoning, a recent meta-analysis found a corrected mean correlation of 0.45 between transformational leadership and affective organizational commitment (Jackson et al., 2013).

Past studies have consistently found positive associations between affective organizational commitment and OCB (Ng and Feldman, 2011). Besides social exchange processes, this relationship can be partly explained because organizational commitment comprises an affective component, and the experience of positive affect makes people more likely to engage in OCB (George and Brief, 1992). Other arguments to support a positive relationship between transformational leadership and OCB are largely based on social exchange theory (Cropanzano and Mitchell, 2005). Because social exchange relationships stress the attachments, obligations, and identification that employees feel toward their exchange partner, prior studies have often used affective organizational commitment to operationalize the existence of such a relationship (Cropanzano et al., 2003; Lavelle et al., 2009; Colquitt et al., 2014). That is, to the extent that employees form a social exchange relationship as indicated by high levels of affective commitment, they tend to show more OCB (Cropanzano et al., 2003). Thus, we suggest:

*Hypothesis 2: Affective organizational commitment partially mediates the positive relationship between transformational leadership behaviors and OCB*.

### Transformational leadership behaviors and OCB: a relational mechanism

The second potential mechanism underlying the relationship between transformational leadership behaviors and OCB is a relational process. In addition to elicit positive reactions of followers, transformational leadership behaviors build also interactive relationships with their followers. This notion is probably best exemplified in research linking transformational leadership behaviors with relational constructs such as trust in the leader (Dirks and Ferrin, 2002) and LMX (Wang et al., 2005). Trust in the leader can be referred to as an individual's positive expectations toward the behaviors of the leader and the willingness to become vulnerable to him or her (Rousseau et al., 1998). Transformational leaders build trust by engaging in exemplary acts, which followers interpret as involving personal sacrifice and risk. Additionally, followers are likely to trust their transformational leaders because such leaders advocate their position unselfishly and show a concern for followers' needs (Conger et al., 2000).

Employees who trust their leader are more likely to show OCB. Mayer et al's (1995) influential trust model proposes that followers draw inferences about their leader's trustworthiness, and those inferences influence attitudes and behavior. For example, the model suggests that followers are more likely to engage in behaviors that put them at risk (e.g., proactive engagement, sharing sensitive information) when they think their leader has high integrity, capability, and/or benevolence. In contrast, when followers do not trust their leader (e.g., because of a lack of integrity), they will allocate resources toward “covering their backs,” preventing them from engaging in extra-role behavior such as OCB (Dirks and Ferrin, 2002). In line with this reasoning, a recent meta-analysis found a corrected mean correlation of 0.48 for the relationship between trust in the leader and OCB (Colquitt et al., 2013). Past work supports the mediating role of trust in the relationship between transformational leadership and employee OCB. For example, integrating the literature on trust in leadership, Burke et al. (2007) provide propositions that link transformational leadership to trust and trust to OCB. In another study, Pillai et al. (1999) built on social exchange theory and found an indirect relationship of transformational leadership with OCB through trust in the leader. Based on the theory and evidence presented above, we propose:

*Hypothesis 3: Trust in the leader partially mediates the positive relationship between transformational leadership behaviors and OCB*.

In addition to trust in the leader, another important relational construct is LMX. Based on theories of role making (Graen, 1976) and social exchange, LMX theory proposes that during the various processes of role taking, role negotiation, delegating tasks, and meeting expectations, leaders develop different types of exchange relationships with their followers (Graen and Uhl-Bien, 1995). A low-quality LMX relationship is characterized by instrumental and transactional exchange. A high-quality LMX relationship, however, is characterized by loyalty and reciprocity (Liden and Maslyn, 1998).

The relationship between trust and LMX is complex. Some research considered trust as a subdimension of LMX (for a review see Schriesheim et al., 1999); other studies empirically or conceptually separated the two constructs (e.g., Cunningham and MacGregor, 2000). According to Brower et al. (2000) one of the main differences is that LMX theory treats the quality of the exchange relationship as an objective phenomenon, whereas trust is a subjective perception held by the trustor. Additionally, they point out that the notion of risk is central to the definition of trust but unrelated to LMX theory. Given the lack of consensus regarding the relationship between trust and LMX, Dirks and Ferrin (2002) considered trust in leadership and LMX as correlated but distinct constructs. Thus, it is reasonable to treat trust and LMX as distinct constructs, and at the same time, as specific reflections of a general relational factor.

The quality of the LMX relationship affects important leader and member attitudes and behaviors, such as job performance and OCB (Dulebohn et al., 2012). In contrast to transformational leadership referring to leader behaviors, LMX describes the relationship between a leader and a follower which is only partly a consequence of leader behaviors (Dulebohn et al., 2012). In contrast to job attitudes such as job satisfaction referring to a more general evaluation of one's job, LMX focuses on the perceived quality of a specific relationship between a leader and a follower. Moreover, job attitudes comprise mainly one-directional reactions of followers to leader behaviors, whereas LMX includes bi-directional relations between leader and follower characterized by mutual exchange and reciprocity.

Transformational leaders are likely to contribute to a high-quality LMX relationship with their individual followers through inspiring and motivating them and showing concern for their needs. Those leader behaviors may elicit a desire on the part of followers to exert effort in forming high-quality relationships with their leaders (Maslyn and Uhl-Bien, 2001). Thus, followers are likely to experience a high-quality relationship with their leader and feel a sense of obligation to him or her. As a consequence of a high quality relationship with the leader, followers are likely to go beyond required in-role behavior and engage in OCB to reciprocate high LMX relationships and to maintain a balanced social exchange (Ilies et al., 2007). In line with this reasoning, Wang et al. (2005) found support for the mediating role of LMX in the relationship between transformational leadership behaviors and OCB. Thus, we propose:

*Hypothesis 4: LMX partially mediates the positive relationship between transformational leadership behaviors and OCB*.

### Relative strength of the attitudinal vs. relational mechanisms

In the present study, we go beyond examining single mediators in isolation and address the question of whether the attitudinal mechanism (i.e., job satisfaction and affective organizational commitment) and the relational mechanism (i.e., trust in the leader and LMX) are equally strong in mediating the relationship between transformational leadership and OCB or whether the two mechanisms differ in magnitude. For the relationship between transformational leadership and OCB, we are aware of only one study addressing attitudinal and relational mediators in concert. Using a sample of 988 employees and their leaders, Podsakoff et al. (1990) found that trust in the leader mediated the transformational leadership—OCB relationship, whereas follower job satisfaction did not. We go above and beyond this prior study by contrasting a larger set of mediators and using a broader data base.

There are reasons to assume that transformational leadership behaviors are important for triggering the attitudinal path to OCB. For example, transformational leaders who show admirable behaviors which cause followers to identify with them (i.e., idealized influence) and articulate appealing and inspiring visions (i.e., inspirational motivation) are likely to exert a strong influence on affective organizational commitment through orienting followers toward organizational goals and interests. Likewise, transformational leaders who attend to followers' needs and act as mentors should increase followers' job satisfaction through facilitating the achievement of followers' job values.

Similarly, there are reasons to believe that transformational leadership behaviors are important for triggering the relational path to OCB. In general, the fundamental importance of the relational mechanism and the dyadic leader-follower relationship can be inferred from several theories. For example, Baumeister and Leary (1995) proposed that human beings have a fundamental need to belong that is satisfied only by positive and stable interaction patterns with other people. Building on this argument, Smart Richman and Leary (2009) stated that feeling valued and accepted by others leads to a secure sense of relational value. Negative interpersonal experiences, however, can threaten the relational value and elicit behavioral responses such as lowered pro-social behavior and higher withdrawal. Given that employees spend a considerable amount of time at work, their leader is a likely source of relational value and can play an important role in fulfilling employees' relational needs. To contrast the magnitude of the attitudinal and relational mechanisms, we state the following research question:

Research Question 1: Does the attitudinal or the relational mechanism more strongly mediate the relationship between transformational leadership and OCB?

## Methods

### Literature search, inclusion criteria, and coding

We used several search procedures to find relevant meta-analytic correlations. First, we conducted an electronic keyword search within the database *PsycInfo* and the internet search engine *Google Scholar*. Keywords used included the typical terms used to label the six constructs under investigation (i.e., transformational leadership, OCB, affective organizational commitment, job satisfaction, trust in the leader, and LMX). To restrict the literature search to meta-analyses, we combined these keywords with the additional terms *quantitative review* or *meta-analysis*. Second, we inspected the reference lists of previous meta-analyses to identify correlations relevant to our study. Finally, we conducted a hand search in the following journals: *Academy of Management Journal, Journal of Applied Psychology, The Leadership Quarterly, Journal of Management, and Personnel Psychology*. Given that only comprehensive and relatively new meta-analysis would improve the set of meta-analyses already identified through the other search strategies, the hand search focused on articles published within the last 5 years (till February 2017). The literature search was conducted in October 2014 and updated in February 2017.

In some cases, more than one meta-analysis provided a correlation for the same relationship. In this case, we used the correlation with the largest sample size. Additionally, we used correlations corrected for measurement error, because our interest lies in construct-level relations. For all relationships involving OCB, we relied on non-self-report measures of OCB because self-ratings might have contained inflation or other biases. The only exception is the correlation between trust in the leader and OCB which is based on a mixture of non-self-reported and self-reported OCB. Because we are unaware of a meta-analysis that examined the relationship between trust in the leader and affective organizational commitment, we used a correlation between trust in the leader and overall organizational commitment from Dirks and Ferrin (2002). Table 1 provides the 15 correlations used to test our hypotheses.

**Table 1:** Meta-analytic correlations.

	**1**	**2**	**3**	**4**	**5**
1. Transformational leadership	–				
2. OCB (non-self-report)	0.27 (48, 11,766)^a^	–			
3. Affective org. commitment	0.44 (100, 34,873)^a^	0.22 (64, 17,509)^b^	–		
4. Job satisfaction	0.48 (84, 32,667)^a^	0.24 (69, 17,672)^c^	0.65 (69, 23,656)^d^	–	
5. Trust in the leader	0.67 (26, 9,491)^a^	0.27 (12, 3,002)^e^	0.59 (40, 9,676)^f^	0.65 (34, 10,631)^e^	–
6. LMX	0.75 (26, 9,246)^a^	0.31 (72, 15,365)^g^	0.41 (21, 8,118)^h^	0.49 (88, 22,520)^h^	0.65 (8, 1,217)^g^

*Table contents: r_c_ (k, N). OCB, organizational citizenship behavior; LMX, leader-member exchange; org., organizational; r_c_, corrected population correlation; k, number of studies; N, cumulative sample size*.

a *Ng (2017)*.

b *Ng and Feldman (2011)*.

c *Correlation from Ilies et al. (2009)*.

d *Correlation from Meyer et al. (2002)*.

e *Colquitt et al. (2007), mix of supervisor- and self-reported OCB*.

f *Correlation from Dirks and Ferrin (2002)*.

g *Martin et al. (2016)*.

h *Dulebohn et al. (2012)*.

We coded effect sizes, sample sizes, and number of studies. The sum of all sample sizes of the 15 correlations included in the present study was 227,419 (mean = 15,161, min = 3,002, max = 34,873). Total number of studies was 761 (mean = 51, min = 8, max = 100).

### Analysis

We performed a set of meta-analytic path analyses (Viswesvaran and Ones, 1995). For these computations, we used sample-size-weighted mean correlations corrected for unreliability from published meta-analyses (see Table 1). The software Mplus 7.2 (Muthén and Muthén, 2012) with maximum likelihood estimation was used for these analyses. In line with prior meta-analyses (e.g., Colquitt et al., 2013), we used the harmonic mean sample size to compute the standard errors for the path coefficients. This practice gives less weight to large samples and results in more conservative estimates compared to the average or the sum of the studies' sample sizes. To examine hypotheses 1 to 4, we tested four meta-analytic path models separately for the four potential mediators affective organizational commitment, job satisfaction, trust in the leader, and LMX. To compare full vs. partial mediation, we used the log-likelihood ratio test. Chi-square based model fit indices (e.g., Tucker Lewis Index, Comparative Fit Index, and Root Mean Square Error of Approximation) could not be used for model comparisons, because the full mediation model does not provide chi-square model fit indices because it is fully saturated. To examine the relative strength of the mediators (Research Question 1), we tested a multiple mediation model comprising all mediators simultaneously. In this model, relationships among the mediators were allowed to be freely estimated. For the direct relationships, we report unstandardized path coefficients and their 95% confidence intervals (CI). For the mediation results, we use the product-of-coefficients method to obtain point estimates and their 95% CI.

## Results

### Attitudinal mechanism

Table 2 shows results of meta-analytic path analyses. Hypothesis 1 predicts that job satisfaction partially mediates the positive relationship between transformational leadership and OCB. A model with a direct path from transformational leadership to OCB fit the data better than a model without this path [Δ-2 × LL (1) = 585.42, *p* < 0.01]. Results revealed a significant indirect effect (unstandardized estimate of the product-of-coefficients = 0.07, *p* < 0.01, 95% CI = 0.06, 0.08) and a significant direct relationship between transformational leadership and OCB (*b* = 0.20, *p* < 0.01, 95% CI = 0.19, 0.22). Thus, results supported Hypothesis 1.

**Table 2:** Results of single mediation models.

	**Δ–2 ×0020LL (Δdf)**	**a**	**b**	**c‘**	***R*^2^**	**Δ*R*^2^**	**Indirect effect**	***N* (harmonic mean)**
TFL → job satisfaction → OCB	585.42^**^ (1)	0.48^**^	0.14^**^	0.20^**^	0.09	0.02	0.07^**^	17,424
TFL → com → OCB	700.52^**^ (1)	0.44^**^	0.13^**^	0.22^**^	0.09	0.02	0.06^**^	17,567
TFL → LMX → OCB	41.39^**^ (1)	0.75^**^	0.25^**^	0.09^*^	0.10	0.03	0.18^**^	11,618
TFL → trust in leader → OCB	89.75^**^ (1)	0.67^**^	0.16^**^	0.16^**^	0.09	0.02	0.11^**^	5,731

TFL, transformational leadership; com, affective organizational commitment; OCB, organizational citizenship behavior; LMX, leader-member exchange; LL, log-likelihood; Δ-2 × LL, model comparison between models with and without a direct TFL → OCB path (significant Δ–2 × LL values indicate that the model with a direct TFL → OCB path fits the data better); df, degrees of freedom; a, TFL → mediator path; b, mediator → OCB path; c‘, TFL → OCB path when the mediator is included in the model (i.e., direct effect); R^2^, amount of variance explained in OCB; ΔR^2^, change in explained variance when the mediator is added to the model compared to the total effect of TFL on OCB (c-path = 0.27, R^2^ = 0.07); N, number of individuals.

* p < 0.05,

** *p < 0.01*.

Hypothesis 2 proposes that affective organizational commitment partially mediates the positive relationship between transformational leadership and OCB. Model comparisons revealed that a model with a direct path from transformational leadership to OCB fit the data better than a model without this path [Δ-2 × LL (1) = 700.52, *p* < 0.01]. Additionally, results showed a significant indirect effect (unstandardized estimate of the product-of-coefficients = 0.06, *p* < 0.01, 95% CI = 0.05, 0.06) and a significant direct relationship between transformational leadership and OCB (*b* = 0.22, *p* < 0.01, 95% CI = 0.20, 0.23) when affective organizational commitment was entered as mediator into the model. Those results suggest that affective organizational commitment partially mediates the relationship between transformational leadership behaviors and OCB. Thus, the data supported Hypothesis 2.

### Relational mechanism

Hypothesis 3 predicts that trust in the leader partially mediates the positive relationship between transformational leadership and OCB. Model comparisons indicated that a model with a direct path from transformational leadership to OCB fit the data better than a model without this path [Δ–2 × LL (1) = 89.75, *p* < 0.01]. Additionally, results supported the mediating role of trust in the leader, as indicated by a significant indirect effect (unstandardized estimate of the product-of-coefficients = 0.11, *p* < 0.01, 95% CI = 0.09, 0.13) and a significant direct relationship between transformational leadership and OCB (*b* = 0.16, *p* < 0.01, 95% CI = 0.13, 0.20) when trust in leader was entered as mediator into the model. Thus, results revealed partial mediation and supported Hypothesis 3.

Hypothesis 4 proposes that LMX partially mediates the positive relationship between transformational leadership behaviors and OCB. A model with a direct path from transformational leadership to OCB fit the data better than a model without this path [Δ–2 × LL (1) = 41.39, *p* < 0.01]. Results showed a significant indirect effect (unstandardized estimate of the product-of-coefficients = 0.18, *p* < 0.01, 95% CI = 0.16, 0.20) and a significant direct relationship between transformational leadership and OCB (*b* = 0.09, *p* < 0.01, 95% CI = 0.06, 0.11) when LMX was entered as mediator into the model. Thus, the data supported Hypothesis 4.

### Attitudinal vs. relational mechanisms

**Figure 1:**
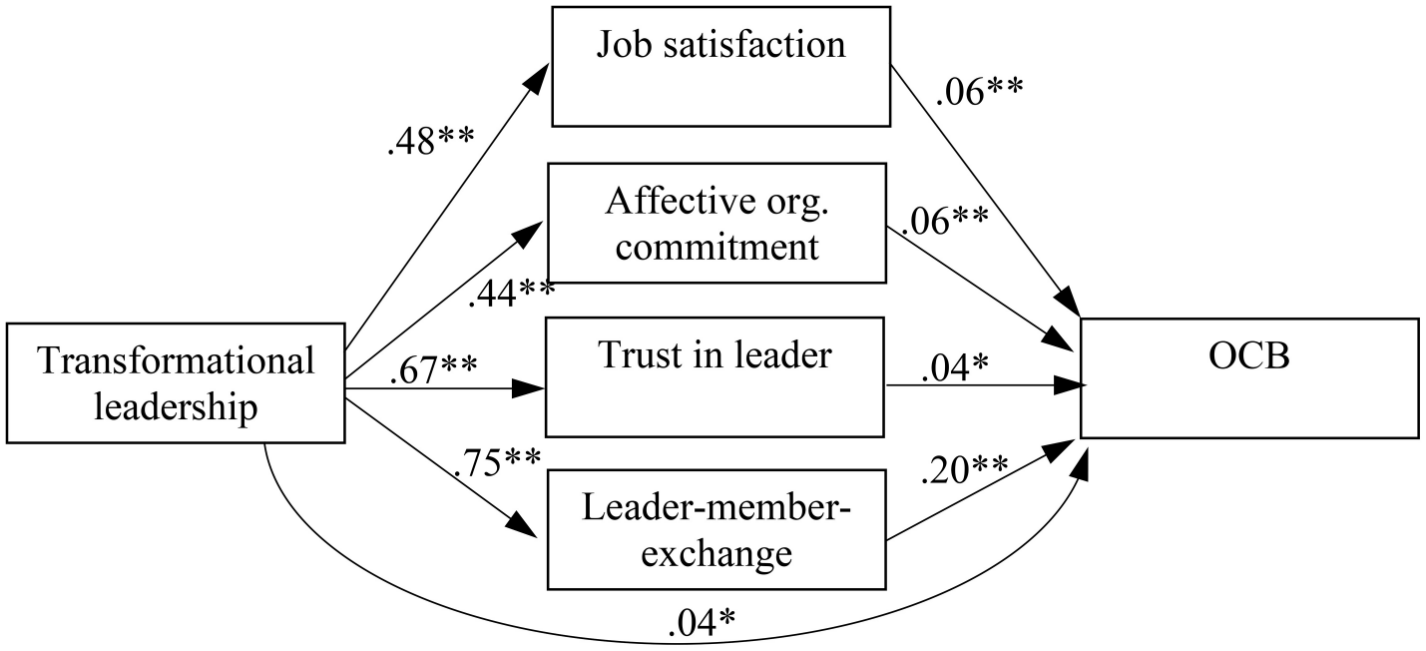
Results of meta-analytic path analyses. *N* = 7,148 (harmonic mean); OCB, organizational citizenship behavior; org., organizational; ^*^p < 0.05; ^**^p < 0.01.

To examine whether the attitudinal or the relational mechanism more strongly mediates the relationship between transformational leadership and OCB (Research Question 1), we simultaneously entered job satisfaction, affective organizational commitment, trust in the leader, and LMX into a multiple mediator model (see Figure 1). Model comparisons revealed that a model with a direct path from transformational leadership to OCB fit the data better than a model without this path [Δ–2 × LL (1) = 3.91, *p* < 0.05]. Results showed that the indirect effect via LMX (unstandardized estimate of the product-of-coefficients = 0.15, *p* < 0.01, 95% CI = 0.13, 0.18) was stronger than the indirect effects via job satisfaction (unstandardized estimate of the product-of-coefficients = 0.03, *p* < 0.01, 95% CI = 0.01, 0.04) and affective organizational commitment (unstandardized estimate of the product-of-coefficients = 0.03, *p* < 0.01, 95% CI = 0.01, 0.04), as indicated by significant contrasts between the indirect effects (for LMX vs. job satisfaction: unstandardized estimate of the contrast = 0.13, *p* < 0.01, 95% CI = 0.09, 0.16; for LMX vs. commitment: unstandardized estimate of the contrast = 0.13, *p* < 0.01, 95% CI = 0.10, 0.16). Thus, the first contrasts provided preliminarily support for the notion that relational constructs are the stronger mediators compared to attitudinal constructs.

However, the indirect effect via trust in the leader (unstandardized estimate of the product-of-coefficients = 0.03, *p* < 0.05, 95% CI = 0.003, 0.05) was similar in magnitude as the indirect effects via job satisfaction and affective commitment, as indicated by non-significant contrasts (for trust in leader vs. job satisfaction: unstandardized estimate of the contrast = 0.00, *n.s.*, 95% CI = −0.03, 0.03; for trust in leader vs. commitment: unstandardized estimate of the contrast = 0.00, *n.s*., 95% CI = −0.03, 0.03). So far, results lend only arguably support for relational constructs (trust in the leader and LMX) as being stronger mediators compared to attitudinal constructs (affective organizational commitment and job satisfaction).

**Figure 2:**
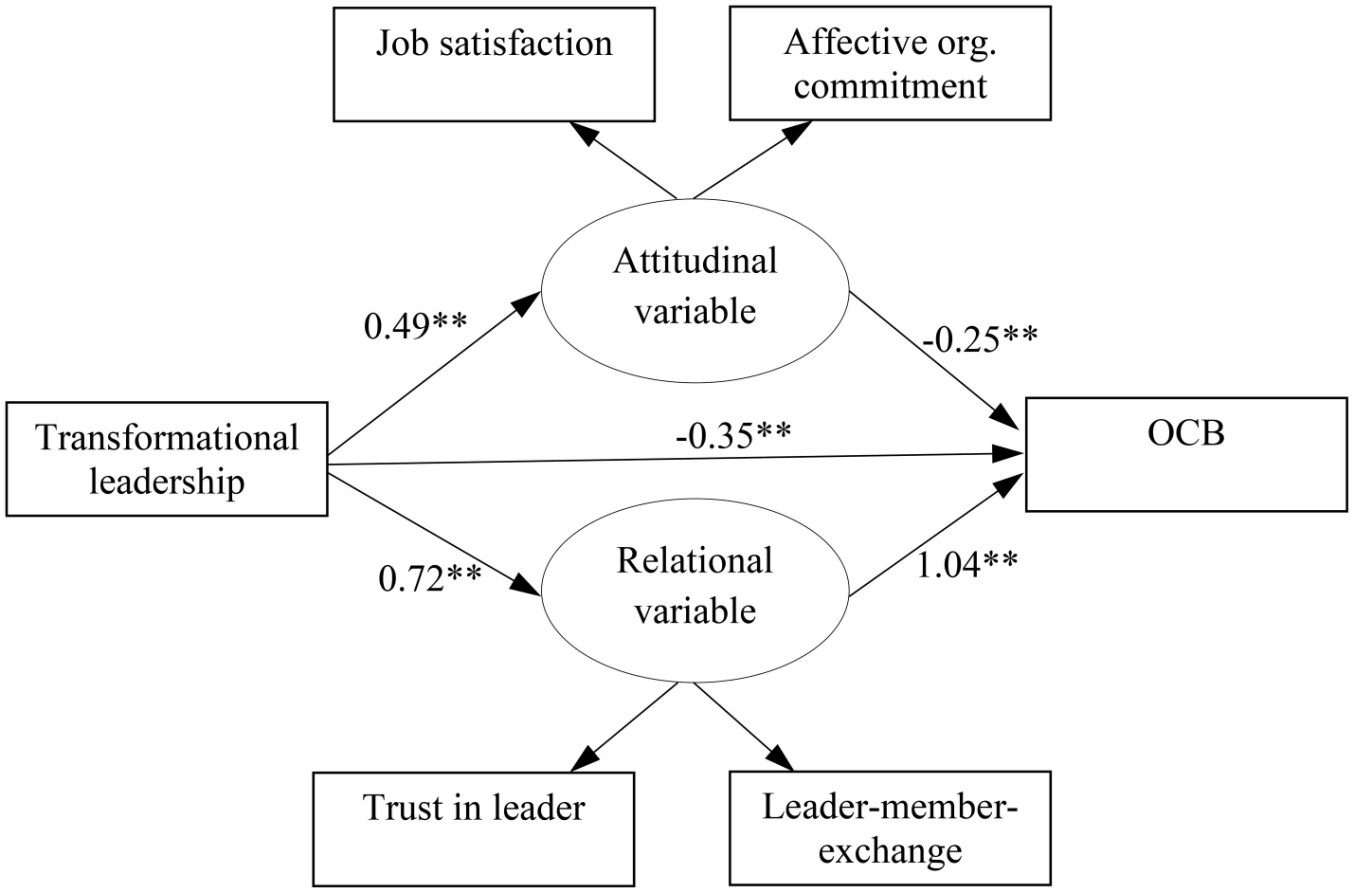
Results of meta-analytic path analyses. *N* = 7,148 (harmonic mean); OCB, organizational citizenship behavior; org., organizational; ^**^p < 0.01.

To capture the attitudinal and relational mechanisms on a more theoretical, higher-order level, we performed additional analyses using a latent attitudinal and a latent relational variable (Figure 2). Specifically, job satisfaction and affective commitment served as indicators for the latent attitudinal variable and trust in the leader and LMX served as indicators for the latent relational variable. Residuals of the latent attitudinal and the latent relational variables were allowed to vary freely. Because latent variables require multiple indicators and we had only one indicator each for transformational leadership and OCB, we could not use latent variables for those two constructs. We favored a partial mediation model including a direct path from transformational leadership to OCB because it had a better fit to the data compared to a full mediation model without such a direct path [Δ-2 × LL (1) = 29.65, *p* < 0.01]. Figure 2 shows results of the partial mediation model. Results revealed that the indirect effect via the relational mechanism (unstandardized estimate of the product-of-coefficients = 0.74, *p* < 0.01, 95% CI = 0.51, 0.97) was stronger than the indirect effect via the attitudinal mechanism (unstandardized estimate of the product-of-coefficients = −0.12, *p* < 0.01, 95% CI = −0.20, −0.05) as indicated by a significant contrast (unstandardized estimate of the contrast = 0.62, *p* < 0.01, 95% CI = 0.46, 0.77). Interestingly, transformational leadership (b = −0.35, *p* < 0.01, 95% CI = −0.50, −0.19) and the attitudinal variable (b = −0.25, *p* < 0.01, 95% CI = −0.41, −0.10) were negatively related to OCB. The meaning of the attitudinal variable must be seen in the context of the other variables included in the model. Specifically, controlling for the relational variable when regressing OCB on the attitudinal variable results in a job attitude—OCB relationship that lacks relational aspects. In this context, the variance in the attitudinal variable that is uncorrelated with the relational variable shows a negative relationship with OCB (i.e., suppression effect). Thus, on a more abstract level, results revealed that the relational mechanism more strongly mediated the relationship between transformational leadership and OCB compared to the attitudinal mechanism (Research Question 1).

### Additional analyses: alternative model

One might argue that the variables examined in this study should occupy different positions than postulated in our hypothesized model. In an alternative model, transformational leadership behaviors could predict relational constructs (i.e., trust and LMX), which predict job attitudes (i.e., satisfaction and commitment), which predict OCB (“sequence” model). Specifically, in this alternative sequence model, we specified relationships (i) from transformational leadership to trust, LMX, and OCB, (ii) from trust and LMX to both satisfaction and commitment, and (iii) from satisfaction and commitment to OCB. Model comparisons revealed that the hypothesized model showed a better fit to the data than the sequence model [Δ–2 × LL (4) = 182.08, *p* < 0.01]. Additionally, we performed model comparisons using a latent attitudinal variable with job satisfaction and affective commitment as indicators and a latent relational variable with trust in leader and LMX as indicators. Again, we compared the hypothesized model (Figure 2) to the sequence model (i.e., transformational leadership → latent relational variable → latent attitudinal variable → OCB; and transformational leadership → OCB). Again, the hypothesized model showed a better fit to the data compared to the sequence model [Δ–2 × LL (2) = 526.08, *p* < 0.01]. Thus, the rejection of the sequence model strengthens confidence in the results of the hypothesized model.”

## Discussion

Using meta-analytic path analyses, this study examined four potential mediators of the positive relationship between transformational leadership behaviors and OCB: Job satisfaction, affective organizational commitment, trust in the leader, and LMX. When tested individually, results supported the assumption that each of them mediated the relationship between transformational leadership behaviors and OCB. When tested simultaneously, results revealed that LMX is the stronger mediator compared to the two attitudinal constructs job satisfaction and affective organizational commitment. Comparisons of a higher-order attitudinal factor vs. a higher-order relational factor revealed that the relational factor more strongly mediated the relationship between transformational leadership and OCB. Moreover, additional analyses supported our hypothesized model instead of a sequence model in which transformational leadership behaviors predicted relational constructs (i.e., trust and LMX), which predicted job attitudes (i.e., satisfaction and commitment), which predicted OCB.

Our results have various theoretical implications. First, our focus on the role of four potential mediators of the relationship between transformational leadership behaviors and OCB serves to integrate a set of theoretically important mediators and provides insights into their relative strength. Specifically, prior models on the association between transformational leadership and OCB have postulated several mediators of this relationship, such as job satisfaction, trust, or LMX. However, past studies failed to examine whether those mediators are equally strong or whether some of them are stronger than others. Building on the distinction between a relational and an attitudinal mechanism, we found that the relational mechanism is stronger than the attitudinal mechanism. This finding underlines the fundamental importance of transformational leaders in fulfilling employees' relational needs, such as feeling valued and accepted by others. The relational mechanism may be more sensitive to transformational leadership behaviors compared to the attitudinal mechanism, because the relational path reflects a mechanism which is specifically focused on leader behavior, whereas job attitudes reflect a broader and more general mechanism not exclusively focused on leader behavior. For example, a leader who does not show concern for individual followers' needs is unlikely to build trust and LMX with employees. However, followers of such a leader could still be relatively satisfied with their job and feel committed to their organization, because not only leader behaviors influence those job attitudes but also other work-place factors, such as job characteristics and organizational benefits (Loher et al., 1985; Butts et al., 2013). In sum, those results help to better understand the mechanisms underlying the transformational leadership–OCB relationship.

Second, our results corroborate and extend prior work on transformational leadership and OCB. Specifically, our findings add to two recent meta-analyses that examined mechanisms through which leadership is related to performance outcomes. Similar to Ng (2017), our findings support the mediating role of job satisfaction, affective organizational commitment, and LMX. However, Ng (2017) did not address trust in the leader as mediator for the relationship between transformational leadership and OCB. Thus, our findings go beyond this prior study by addressing and supporting trust in the leader as mediator of the transformational leadership—OCB relationship. Similar to Gottfredson and Aguinis (2017), we found that LMX was the strongest mediator compared to job satisfaction, affective organizational commitment, and trust in the leader (notably, Gottfredson and Aguinis used general trust but not trust in the leader). However, Gottfredson and Aguinis (2017) focused on multiple mediation models but did not test single mediation models. Therefore, our findings that job satisfaction, affective organizational commitment, trust in the leader, and LMX individually mediated the transformational leadership—OCB relationship adds additional insights over the results of Gottfredson and Aguinis (2017). Additionally, we go beyond Ng (2017) and Gottfredson and Aguinis (2017) by conceptualizing a higher-order attitudinal and a higher-order relational construct. Thus, we treated job satisfaction and affective organizational commitment as specific reflections of an underlying overall job attitude (Harrison et al., 2006), and trust in the leader and LMX as specific reflections of an underlying overall relationship factor. Our finding that a higher-order relational (vs. attitudinal) mechanism more strongly mediated the relationship between transformational leadership and OCB highlights the importance of a more relationship-based perspective, focusing on the one-on-one relationship between leader and subordinate.

Third, another interesting aspect of our results is that the (remaining) direct effect of transformational leadership on OCB was negative when latent attitudinal and relational variables were used. That is the variance in transformational leadership that is uncorrelated with the attitudinal and relational variables is negatively related to OCB. According to Zhao et al. (2010) this negative relationship may suggest some as-yet-undiscovered negative mediation mechanism. Although, most prior models on transformational leadership focused on positive processes and consequences (Wang et al., 2011), there are first studies addressing the “dark side” of transformational leadership. For example, Kark et al. (2003) found that transformational leadership behaviors were positively associated with follower dependence on the leader. Similarly, scholars suggested that transformational leaders may create a high level of emotional involvement when it is not necessary. As a result, employees may become exhausted over time (Harrison, 1987; Yukl, 1999). Future research could address such negative mechanisms and examine whether some subdimensions of transformational leadership are especially likely to elicit such negative mechanisms. For example, the subdimensions of idealized influence and inspirational motivation could be especially important in creating high levels of dependency and involvement among subordinates. Thereby, future studies would contribute to a more through and complete picture of the relationship between transformational leadership and OCB.

Finally, research on transformational leadership has been criticized for lacking an overarching framework that guides the study of mediation (Van Knippenberg and Sitkin, 2013). We respond to this critique by examining theory-based mechanisms. Specifically, in line with prior work (Pillai et al., 1999) we used social exchange theory as a theoretical lens to study a set of mediators of the relationship between transformational leadership and OCB. Social exchange theory could be further used to guide the study of transformational leadership, mechanisms, and consequences. By more strongly focusing on a single theoretical framework, transformational leadership could become a more coherent field of research. Although, this study and past research on OCB applied social exchange theory mainly to the individual level, it can also be used to develop hypotheses at the team level (Gong et al., 2010). Thus, future research could use social exchange theory to further elaborate mechanisms and consequences of individual- and team-focused transformational leadership behaviors (Wang and Howell, 2010; Chi and Huang, 2014).

### Limitations and implications for future research

This study has of course some limitations. First, although the different subdimensions of transformational leadership are often highly correlated and combined into a single construct (Qu et al., 2015), they could have different mediation patterns (Van Knippenberg and Sitkin, 2013). For example, individual consideration and idealized influence could be especially important for the relational mechanism, whereas inspirational motivation could be especially important for the attitudinal mechanism. Because our study does not differentiate between the subdimensions of transformational leadership, we cannot address this issue. Through specifying and testing how each subdimension influences mediators and outcomes, future studies would enhance our understanding of the relationship between transformational leadership and OCB.

Second, we did not differentiate between different forms of OCB. However, results could unfold differently when different foci of OCB are considered. For example, job attitudes could be especially strong in mediating the relationship between transformational leadership behaviors and OCB directed at the *organization* (OCB-O), and less important in mediating the relationship between transformational leadership behaviors and OCB directed at the *individual* (OCB-I). The notion behind this idea is that employees could be more likely to reciprocate job attitudes in a more general way toward the organization, rather than toward individuals.

Third, because our study is based on cross-sectional correlations, it does not allow causal conclusions. Thus, it would be a fruitful avenue for future research to experimentally test the causal relationships among the constructs examined in the present study.

Fourth, the two mechanisms examined in this study have different referents. That is, job satisfaction and affective organizational commitment have relatively broad referents (i.e., the job and the organization, respectively) whereas trust in the leader and LMX have specific referents (i.e., the leader). Unfortunately, the present study cannot control for potential effects of the different referents. However, whether the different referents affect our findings might be an artificial question because the different referents are closely intertwined with the nature of the constructs under investigation. Of course, to keep the referents constant future studies could use satisfaction with and commitment to the leader instead of job satisfaction and organizational commitment. However, studies using satisfaction with and commitment to the leader are unlikely to answer the question of the relative strength of attitudinal vs. relational mechanisms because satisfaction with and commitment to the leader blur the line between attitudinal and relational constructs.

Finally, transformational leadership has been criticized for being confounded with its effects (Van Knippenberg and Sitkin, 2013). For example, the subdimension of individualized behavior attributed is confounded with outcomes such as respect and trust. Similarly, Shaffer et al. (2016) examined the discriminant validity of several leadership constructs and questioned whether transformational leadership and LMX are empirically distinct. On the other hand, studies report confirmatory factor analyses suggesting the distinctiveness of transformational leadership and outcomes such as trust (Zhu and Akhtar, 2014), LMX (Chun et al., 2016), and affective commitment (Herman et al., 2013). Unfortunately, our study can barely contribute to these ongoing debates; however, it would be an interesting question for future research to determine *the degree* to which confounding affects relationships of transformational leadership with outcome variables. Relatedly, although our mediators are theoretically distinct constructs, they are correlated which might be partly due to conceptual overlap in items used to measure the different mediators. Biased relationships among our mediators due to overlapping items is unlikely to be a major issue in our study, because we tested our mediators in parallel and did not hypothesize direct relationships among them. Indeed, by simultaneously regressing OCB on our four mediators, we account for the shared variance between our mediators and provide a more rigorous test of their mediating role compared to studies testing only single mediators (Wang et al., 2005).

### Implications for organizations

Our results have interesting implications for organizations. Practitioners are well-advised to foster OCB because employee OCB contribute to organizational success (Podsakoff et al., 2009). Because OCB are discretionary behaviors that can be less required by formal job descriptions compared to in-role job performance, it is important to create a stimulating work environment that positively contributes to those voluntary behaviors. In line with prior studies (Wang et al., 2011), our results suggest that transformational leadership behaviors are an important aspect of such a stimulating work environment that contributes to OCB. Thus, organizations should foster transformational leadership behaviors, for example by providing trainings, using suitable selection criteria, and communicating official leadership guidelines (Antonakis et al., 2011).

Additionally, organizations should contribute to an environment in which the positive implications of transformational leadership behaviors can fully unfold. In this regard, our findings offer differential suggestions. Specifically, results of this study suggest that the relational constructs trust in the leader and LMX are especially strong in mediating the relationship between transformational leadership and OCB compared with affective organizational commitment and job satisfaction. Thus, organizations wishing to benefit from transformational leadership's positive implications for OCB should focus on how to best develop important aspects of the dyadic leader-follower relationship such as trust in the leader and LMX. Given the increasing prevalence of e-leadership and geographically distributed work teams, building trust and LMX becomes an even more important challenge to organizations, and organizations might establish additional means (e.g., face-to-face meetings) to enable high quality and trusting relationships between leaders and followers.

What implications follow for affective organizational commitment and job satisfaction? Job attitudes have consistently be found to positively relate to important outcomes such as job performance, turnover, and employee health (Meyer et al., 2002; Riketta, 2008). Thus, organizations are well-advised to emphasize a high-quality relationship between leaders and followers as well as a high level off employee affective organizational commitment and job satisfaction.

## Author contributions

CN and GH designed the study; CN collected and analyzed the data; CN and GH drafted and revised the manuscript; CN and GH gave final approval of the version to be published; and CN and GH agree to be accountable for all aspects of the work.

### Conflict of interest statement

The authors declare that the research was conducted in the absence of any commercial or financial relationships that could be construed as a potential conflict of interest.
